# The mitigating effect of repeated memory reactivations on forgetting

**DOI:** 10.1038/s41539-018-0025-x

**Published:** 2018-04-24

**Authors:** Sydney MacLeod, Michael G. Reynolds, Hugo Lehmann

**Affiliations:** Psychology Department, Trent University, Peterborough, ON Canada

## Abstract

Memory reactivation is a process whereby cueing or recalling a long-term memory makes it enter a new active and labile state. Substantial evidence suggests that during this state the memory can be updated (e.g., adding information) and can become more vulnerable to disruption (e.g., brain insult). Memory reactivations can also prevent memory decay or forgetting. However, it is unclear whether cueing recall of a feature or component of the memory can benefit retention similarly to promoting recall of the entire memory. We examined this possibility by having participants view a series of neutral images and then randomly assigning them to one of four reactivation groups: control (no reactivation), distractor (reactivation of experimental procedures), component (image category reactivation), and descriptive (effortful description of the images). The experiment also included three retention intervals: 1 h, 9 days, and 28 days. Importantly, the participants received three reactivations equally spaced within their respective retention interval. At the end of the interval, all the participants were given an in-lab free-recall test in which they were asked to write down each image they remembered with as many details as possible. The data revealed that both the participants in the descriptive reactivation and component reactivation groups remembered significantly more than the participants in the control groups, with the effect being most pronounced in the 28-day retention interval condition. These findings suggest that memory reactivation, even component reactivation of a memory, makes memories more resistant to decay.

## Memory: Improving memory with “Partial Recall”

No need for total recall, remembering a part can strengthen the whole. When Hugo Lehmann and colleagues at Trent University asked volunteers how many previously seen images contained a simple feature (e.g., living object), forgetting was reduced compared to volunteers who only viewed the images. Furthermore, this benefit was akin to that of volunteers who were asked to described the images in full detail. In both cases, the reduction in forgetting was achieved with only three recall opportunities and persisted for at least 28 days, including improvements in quality and quantity. Thus, our most lasting and vivid long-term memories should be amongst those that we reactivate from time-to-time, whether in whole or in part. This may also account for why some memories are less vulnerable than others to neurodegeneration and brain injury.

## Introduction

Newly learned information goes through a consolidation period, where it is labile and subject to modification, before stabilizing into a lasting memory.^1,2^ It was believed that, once consolidated, the memory became fairly permanent and resistant to change.^3,4^ Now, however, it is well established that a consolidated memory can re-enter a labile state induced by retrieval, a process called memory reactivation.^5^ During this new labile state, the memory becomes susceptible to modification^6–8^ and the reactivation generates a new bout of consolidation (i.e., reconsolidation).^9,10^

Amongst the first neuropsychological demonstrations that reactivated memories could be modified were studies examining the effects of electroconvulsive shock on fear memory.^11–13^ For instance, Misanin et al.^12^ showed that reactivation of a fear memory immediately before electroconvulsive shock treatment caused amnesia for that memory on future retention tests, whereas the memory was unaffected if the electroconvulsive shock treatment was given without prior reactivation. Studies using protein synthesis inhibitors have demonstrated similar effects.^14–17^ Indeed, infusions of anisomycin immediately following a memory reactivation in rats causes amnesic effects, but the same anisomycin treatment fails to impair the memory if not preceded by a reactivation.^17^ Thus, reactivated memories can be disrupted, even potentially erased, following a reactivation.

Memories can also be updated following a reactivation, meaning that new information can be integrated into the original memory during reconsolidation.^18^ In this instance, memories are not being erased or disrupted by exogenous manipulations such as electroconvulsive shock or pharmacological agents, but modified by the presentation of novel information. Hupbach et al.^19^ convincingly demonstrated the updating effect following episodic memory reactivation in humans. Their participants learned a list of objects, followed by a second list of objects 48 h later. When the memory of List 1 was reactivated before learning List 2, their participants reported significantly more List 2 items intrusions in List 1 than their participants who did not receive a reactivation. Hence, when new information was presented immediately following memory reactivation, information from the second list integrated with the original memory. Memory updating is now considered to be a robust phenomenon and has been demonstrated to occur for several types of memories, including episodic and procedural.^19–22^

Reactivation and consequent reconsolidation processes may, in addition to updating, benefit the original memory by increasing its strength,^6,8,23^ a possibility that is gaining substantial scientific interest. Compelling evidence suggests that repeated reactivations increase the neural network that supports a memory, even making it more resistant to brain insult.^24–26^ Other studies have demonstrated that reactivating a memory by brief re-exposure to the acquired information promotes new consolidation bouts that mitigate forgetting by increasing the persistence of the memory and/or its precision.^27–33^

The well-established cognitive psychology phenomenon known as the Testing Effect,^34,35^ in which repeated testing enhances retention performance beyond new study episodes, should likewise be taken as evidence that memory reactivations promote the strengthening of memory during reconsolidation.^7^ The standard procedures used to demonstrate the Testing Effect involve effortful retrieval of the originally studied information and can be accompanied by new studying of that original information.^34,35^ This implies, for instance, that an individual providing a description of the tuxedo they wore at their wedding would benefit the retention of that memory. We often engage, however, in partial or component recall of an event and it is unclear whether this component reactivation elicits a reconsolidation episode capable of strengthening the memory. For example, would an individual mentioning the number of times they have worn a tuxedo also improve their memory for the tuxedo they wore at their wedding? Evidence pertaining to this question is sparse and inconclusive. One study examining reconsolidation in non-human animals suggests that reactivating a memory “linked” to the target memory is insufficient to promote reconsolidation processes in the target memory.^36^ Yet, in another study involving human participants, recall of partial information from a studied text increased memory for that text beyond the elements that were reactivated.^37^ Thus, the benefits of component or indirect reactivations remain unresolved. The focus of this study was to partially elucidate this issue by examining whether repeated component reactivations, and associated reconsolidation processes, could mitigate forgetting in humans similar to what is observed after effortful retrieval in the Testing Effect.

**Fig. 1:**
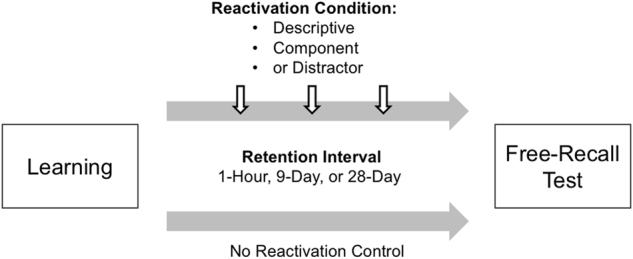
Schematic illustration of the experimental design. The participants briefly viewed a series of neutral images (Learning) and were then assigned to one of three retention intervals: 1 h, 9 days, and 28 days. During these intervals, the participants received three equally spaced memory reactivations. However, the type of reactivations was manipulated and either involved distractor, component, or descriptive reactivations. A no reactivation Control group was also included in the design. At the end, the participants were given an in-lab free-recall test

We assessed the above possibility by having participants reactivate viewed images from the International Affective Picture System (IAPS) using two different approaches: (1) descriptive reactivations, which required the recall of as much information as possible about the viewed images (i.e., detailed written recall of the list of studied images), and (2) component reactivations that cued image categories without requiring detailed feature recall (i.e., provide the number of images that included a category defining attribute). It is well established that repeated testing involving effortful retrieval of information improves retention.^35^ Our descriptive reactivations are analogous to the effortful retrieval procedures in the Testing Effect studies, leading us to predict a reduction in forgetting. Our component reactivations, however, involved partial-reactivation processes that are different than those required at retrieval during the retention test. Importantly, we predicted that this component reactivation, despite the lack of required effortful descriptive retrieval, would also lessen forgetting and demonstrate that reconsolidation processes can increase memory strength. Moreover, we examined the possible benefits of these reactivations over different retention intervals (1 h, 9 days, and 28 days) with the expectation that the reactivation benefits would be the most apparent after the longest retention interval involving the most forgetting. Two control conditions were also included in the study: one condition involved reactivation of procedures without reference to the viewed images (Distractor group), whereas in the other the participants did not receive any reactivation manipulation (Control group). See Fig. 1 for a depiction of the experimental design.

## Results

The participants viewed a total of 26 neutral images and were then given a free-recall test, either 1 h, 9 days, or 28 days later. During the retention interval, the participants were given three equally spaced memory reactivations or no reactivation (Control). The reactivations either involved retrieval of descriptive, component, or distractor information. The dependent variables used to assess memory performance included correctly recalled images, correctly recalled details, and number of correctly recalled details per image, as well as the number of falsely recalled items.

### Correct images

**Fig. 2:**
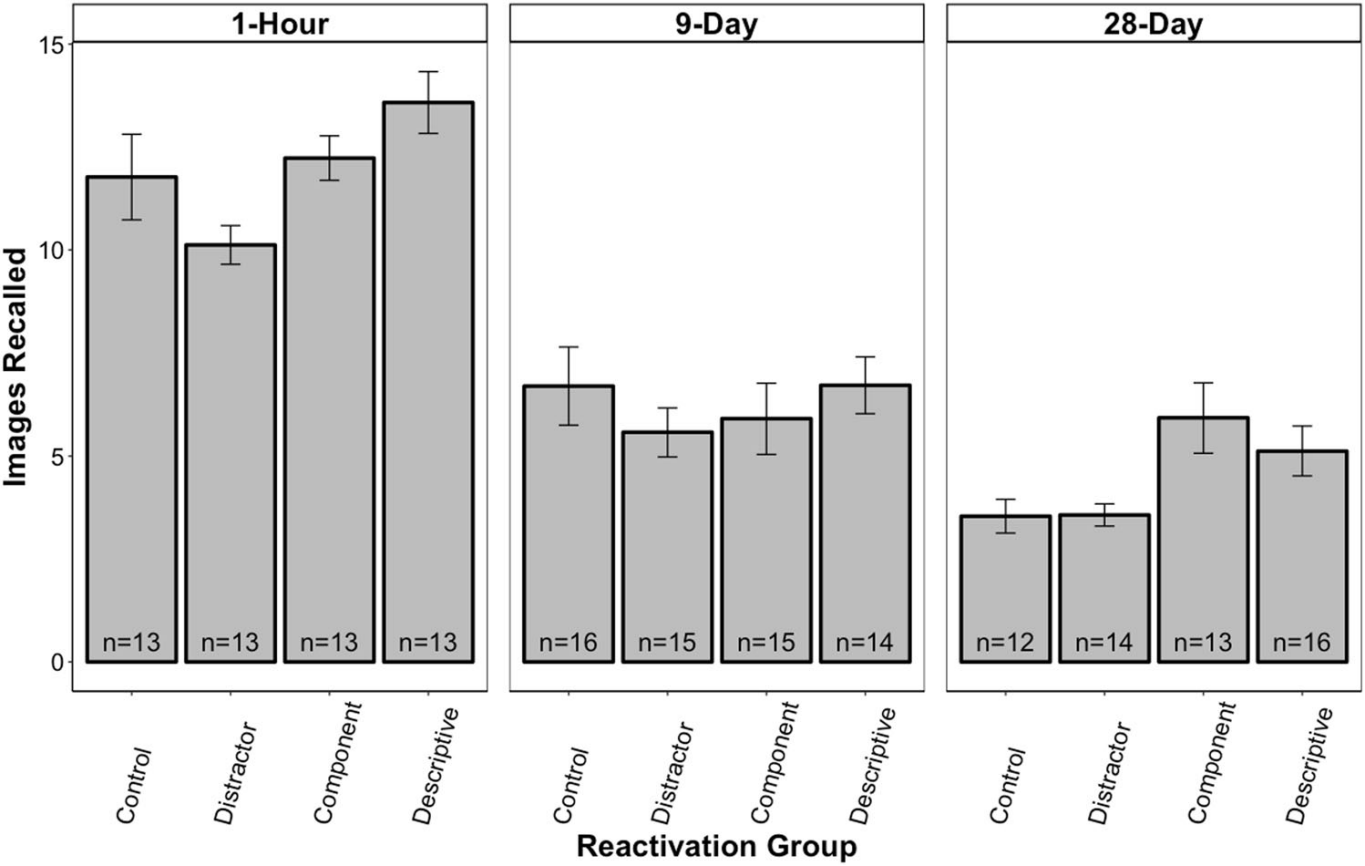
Mean (±SEM) number of correctly remembered images during the free-recall test for each reactivation group across all three retention intervals: 1 h, 9 days, and 28 days. Retention performance significantly decreased across retention intervals (*p*s < 0.05), suggesting time-dependent forgetting. Yet, overall, the participants in the Descriptive and Component groups recalled significantly more images than the participants in the Distractor group (*p*s < 0.05). More strikingly, in the 28-day condition, the participants in both the Descriptive and Component groups recalled significantly more images than the participants in the Distractor and Control groups (*ps* < 0.05), with an relative enhancement exceeding 59%. Hence, the descriptive and component reactivations successfully mitigated forgetting on this index of memory

Figure 2 shows the number of correctly recalled images for each reactivation group across all three retention intervals. The analysis of variance (ANOVA) revealed a significant main effect of interval, *F* (2, 155) = 108.72, *p* < 0.001, a significant main effect of reactivation, *F* (3, 155) = 5.50, *p* = 0.001, but no significant interaction, *F* (6, 155) = 1.29, *p* = 0.263.

The main effect of interval showed a typical time-dependent forgetting curve. Specifically, least significant difference (LSD) pairwise comparisons revealed that the participants in the 28-day condition remembered significantly fewer images than the participants in the 9-day condition (*p* < 0.01), and the 1-h condition (*p* < 0.001). The participants in the 9-day condition also remembered significantly fewer images than the participants in the 1-h condition (*p* < 0.001).

When examining the Reactivation main effect, it was found, as predicted, that the participants in the Descriptive group remembered significantly more images than the participants in the Distractor group (*p* < 0.001) and the no reactivation Control group (*p* < 0.05), demonstrating mitigated forgetting. The component reactivations also mitigated forgetting as the participants in this group remembered significantly more images than the participants in the Distractor group (*p* < 0.01). However, the Component group did not perform significantly better than the no reactivation Control group (*p* = 0.25). Note that the Distractor group and the no reactivation Control group did not significantly differ (*p* = 0.13).

### Correct details

**Fig. 3:**
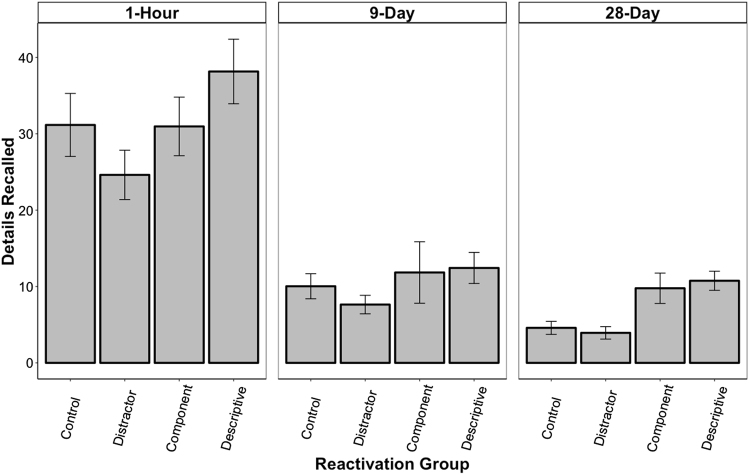
Mean (±SEM) number of correctly remembered details during the free-recall test for each reactivation group across all three retention intervals: 1 h, 9 days, and 28 days. Retention performance significantly decreased across retention intervals (*p*s < 0.05), again suggesting forgetting over time. However, overall, both the Descriptive and Component groups recalled significantly more details than the participants in the Distractor group (*p*s < 0.05). At the longest retention interval, the participants in both the Descriptive and Component groups recalled significantly more details than the participants in both the Distractor and Control groups (*p*s < 0.05) and the relative retention enhancement exceeded 100%. Thus, the descriptive and component reactivations successfully mitigated forgetting on this memory measure as well

Figure 3 shows the average number of correctly recalled details for each reactivation group across all three retention intervals. The ANOVA revealed a significant main effect of Interval, *F* (2, 155) = 89.07, *p* < 0.001, and a significant main effect of reactivation, *F* (3, 155) = 5.24, *p* = 0.002, but no interaction, *F* (6, 155) = 0.64, *p* *=* 0.70.

Again, consistent with time-dependent forgetting, LSD pairwise comparisons revealed that the participants in the 28-day and 9-day conditions remembered significantly fewer details than the participants in the 1-h condition (*p*s < 0.001). The performance of the participants in the 28-day condition, despite a tendency, did not significantly differ from those in the 9-day condition (*p* = 0.09).

When examining the Reactivation main effect, it was found that the participants in the Descriptive group remembered significantly more details than the participants in the Distractor group (*p* < 0.001) and the no reactivation Control group (*p* < 0.05), demonstrating mitigated forgetting. The participants in the Component group remembered more details than the Distractor group (*p* < 0.05), but did not significantly differ from the no reactivation Control group (*p* = 0.31). The Descriptive group did not significantly differ from the Component group (*p* = 0.19).

### Correct details per image

**Fig. 4:**
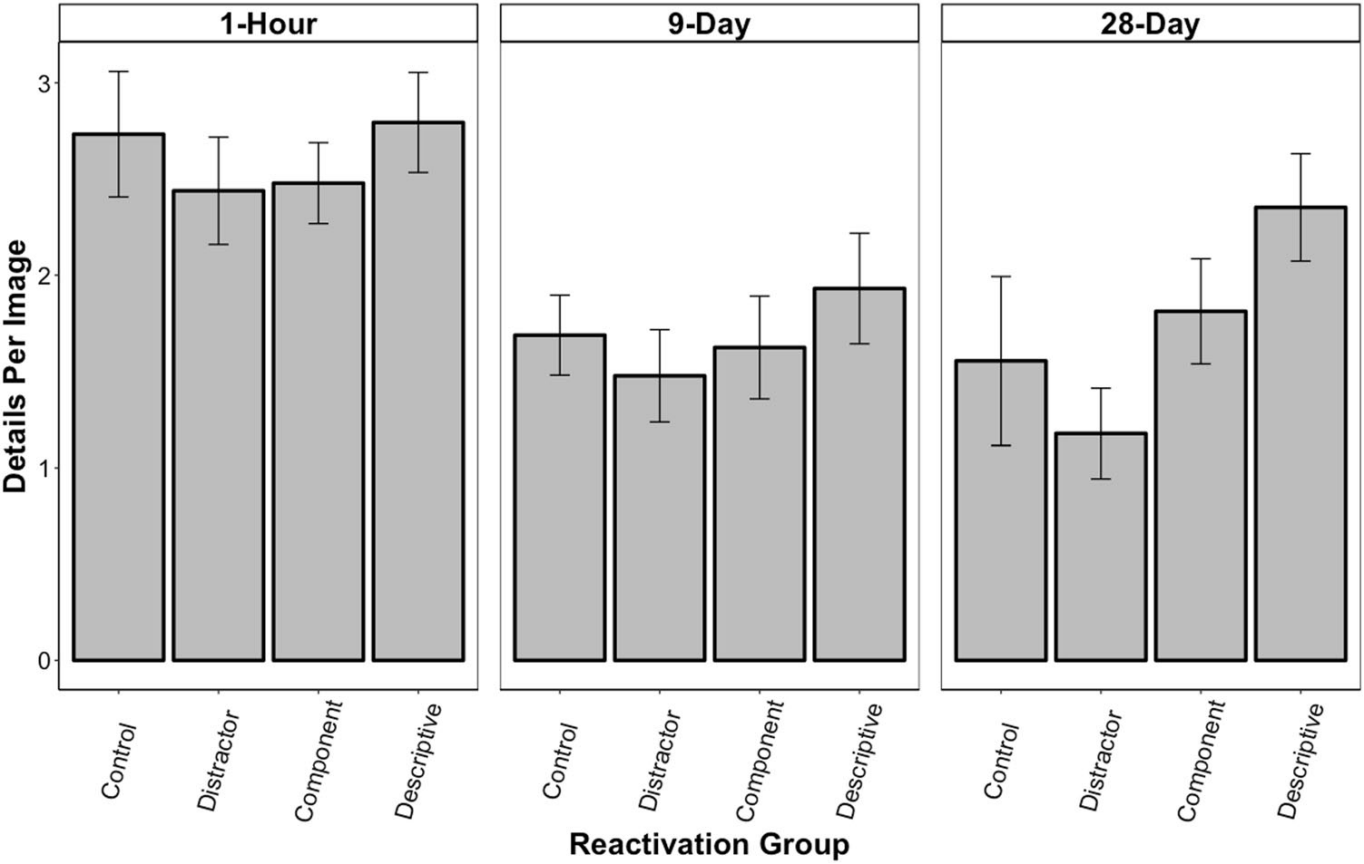
Mean (±SEM) number of correctly remembered details per recalled image during the free-recall test for each reactivation group across all three retention intervals: 1 h, 9 days, and 28 days. This index is more revealing of the quality/precision of the memory for each recalled image. In the 28-day condition, the participants in the Descriptive group recalled significantly more details per image than the participants in the Distractor and Control groups (*p*s < 0.05), a finding that was not observed in the Component group. Hence, only the descriptive reactivation improved precision retention

The total number of details recalled may be confounded with the number of images recalled, therefore Fig. 4 shows the average number of details recalled per correctly recalled image for each group across all three retention intervals. This was conducted to determine whether the reactivation method increased the quality of the retained mnemonic representation. The ANOVA revealed a significant main effect of interval, *F* (2, 155) = 13.97, *p* < 0.001, and a significant main effect of reactivation, *F* (3, 155) = 2.94, *p* = 0.04, but no interaction, *F* (6, 155) = 0.59, *p* *=* 0.74.

Consistent with time-dependent forgetting, LSD pairwise comparisons revealed that the participants in the 28-day and 9-day conditions remembered significantly fewer details per image than the participants in the 1-h condition (*p*s < 0.001). The performance of the participants in the 28-day condition did not significantly differ from that of the participants in the 9-day condition (*p* = 0.82).

When examining the Reactivation main effect, it was found that the Descriptive group remembered significantly more details per image than the Distractor group (*p* < 0.01). There were no other significant differences amongst the other groups (*p*s > 0.05).

### False recall

Table 1 provides the descriptive statistics for the number of falsely recalled images as well as the number of falsely recalled details. These errors were analyzed to determine whether reactivations caused intrusion or added noise to the mnemonic representations.

**Table 1:** Descriptive statistics for falsely recalled images and details

Group	Condition	Mean	SD
False images			
Control	1-h	0.08	0.28
	9-day	2.75	4.67
	28-day	3.00	3.52
Distractor	1-h	0.23	0.44
	9-day	1.33	1.18
	28-day	2.79	2.94
Component	1-h	0.15	0.38
	9-day	1.93	1.49
	28-day	1.77	2.56
Descriptive	1-h	0.08	0.28
	9-day	0.79	1.12
	28-day	2.5	3.27
False details			
Control	1-h	1.92	2.10
	9-day	1.94	1.80
	28-day	1.16	1.19
Distractor	1-h	1.00	1.47
	9-day	1.40	1.50
	28-day	1.50	1.40
Component	1-h	1.46	1.81
	9-day	1.4	2.06
	28-day	1.31	1.11
Descriptive	1-h	1.15	1.63
	9-day	1.21	1.12
	28-day	2.00	1.79

When examining the number of false images reported, the ANOVA revealed a significant main effect of Interval, *F* (2, 155) = 13.57, *p* < 0.001. The participants in the 1-h condition reported significantly fewer false images than the participants in the 9-day condition (*p* < 0.001) or the 28-day condition (*p* < 0.001). There were no significant differences between the 9-day condition and 28-day condition (*p* = 0.07). Neither the main effect of reactivation, *F* (3, 155) = 0.40, *p* = 0.76, nor the interaction, *F* (6, 155) = 0.77, *p* = 0.59, approached significance.

When examining the number of falsely recalled details, the ANOVA failed to reveal any significant effects (interval, *F* (2, 155) = 0.08, *p* = 0.93; reactivation, *F* (3, 155) = 0.40, *p* = 0.76; and interaction, *F* (6, 155) = 0.82, *p* = 0.56).

### 28-Day condition analysis

It was predicted that the mitigating effect of reactivations would be the most pronounced at the longest time interval as this is where participants would experience the highest likelihood of forgetting. These data were, therefore, analyzed independently of the other retention intervals using pairwise comparisons to examine group differences. Both the Descriptive and the Component groups remembered significantly more images than the Distractor group (*p*s < 0.05) and the no reactivation Control group (*p*s < 0.05). Similarly, the Descriptive and Component groups remembered significantly more correct details than the Distractor and no reactivation Control groups (*ps* < 0.05). These findings suggest that both types of reactivation mitigated forgetting. The Descriptive and Component groups did not significantly differ from each other (*p*s > 0.05) nor did the Distractor and no reactivation Control groups (*p*s > 0.05) on either of the two measures. In addition, the participants in the Descriptive group remembered significantly more details per image than participants in the Distractor group (*p* < 0.05). There was no effect of reactivation group on number of false images or false details recalled (*p*s > 0.05).

To better depict the magnitude of the reactivation benefits on retention, we transformed the absolute retention score of each participant in the Descriptive and Component groups into a relative score using the no reactivation Control group average (change relative to Control). This revealed that image recall was enhanced by 58.8% (SEM = 18.4) for the Descriptive group and 67.2% (SEM = 23.9) for the Component group. Similarly, for both groups the number of details recalled increased by 134.5% (SEM = 27.34) and 113.1% (SEM = 43.4), respectively. The correct details per image was also increased by 44.5% (SEM = 19.4) for the Descriptive group, but only by 16.6% (SEM = 17.6) for the Component group. All these increases were significantly above what would be expected by chance (*p*s < 0.05), with the exception of the Component group on the correct details per image measure (*p* > 0.05). Importantly, the Distractor group did not have any significant relative increases in performance on these measures (*p*s > 0.05). Actually, all relative changes were negative for this group.

## Discussion

The current study examined whether memory reactivations strengthen memory as indicated by mitigated forgetting. Importantly, the effects of repeated memory reactivations on long-term retention were assessed using two different approaches: descriptive and component reactivations. The descriptive reactivations required recall of as much information as possible about the viewed images. The component reactivations, on the other hand, required partial recall of categorical features of the previously viewed images, with no detailed description. Both types of reactivations mitigated forgetting. The benefits of the reactivations, however, were the most prominent at the 28-day interval when the memories were the weakest. In this instance, image recall, relative to control performance, was increased by 59% and 67%, respectively, for the Descriptive and Component groups. The relative effect was even greater for the number of recalled details with an increase exceeding 100% for both reactivation groups. Thus, these findings confirm the view that reactivations strengthen memories.

The beneficial memory strengthening effects following the descriptive reactivations are consistent with those of numerous studies that have examined the Testing Effect, in which detailed effortful retrieval of previously studied information results in improved retention.^34,35^ Importantly, our component reactivation findings add to this literature by demonstrating that cueing category retrieval also benefits the memory. In addition, the component findings may elucidate some of the debated mechanisms supporting the Testing Effect. Transfer appropriate processing has been proposed as one possible explanation for the Testing Effect.^38^ This perspective suggests that effortful reactivation-induced retrievals must be similar in form to the final test to promote better retention performance. Although the results of our Descriptive group support this view, the component reactivation findings, in which the reactivation process differs from that of the final test, do not. The component findings suggest that promoting reactivation of the target memories, even in a different retrieval form, is sufficient to promote long-term retention. Our combined descriptive and component reactivation findings may provide better support for theories such as the Elaborative Retrieval Hypothesis or the Construction-Integration Model, which posits that retrieval, regardless of setting and features, causes widespread network activation that increases the number of traces supporting the memory and/or promotes more pathways to it.^34,39,40^ With this greater network, access to the target memories at the time of testing is facilitated and benefits retention performance.^39^

Our findings also extend our knowledge about the Testing Effect on other fronts. First, our demonstration that reactivations can improve memory by mitigating forgetting up to a month after the initial learning goes beyond the typical retention intervals, of hours to a few days, examined in most studies.^35,41,42^ Second, learning only occurred once without the possibility of additional study in our experimental design, whereas other studies often intermix new study episodes with their reactivations.^41,43,44^ Third, the Testing Effect is typically demonstrated using word lists,^45^ facts,^46^ word pairs,^34,39^ prose,^47^ and course material.^41,43,44^ Here, we show that the benefits of reactivations on long-term retention also applies to visual stimuli, such as pictures of scenes without the use of verbal information.^48^

We believe that the reactivations in the current study, whether descriptive or component, strengthened long-term memory (28 days) by initiating cellular/synaptic reconsolidation bouts. When a memory is initially consolidated, it undergoes protein synthesis resulting in neuronal changes that convert the memory to long-term storage.^49,50^ This process, called cellular consolidation, takes place hours after an initial learning period and strengthens the memory trace. Despite the original belief that this was a one-time process,^3,4^ it has been convincingly demonstrated that each time a memory is reactivated it undergoes a new bout of consolidation.^5^ Though these reconsolidation bouts involve plastic changes that differ from those of the initial consolidation,^15,51^ they contribute to memory maintenance.^9,52^ In contrast, the reactivation-induced memory enhancement within the 1-h condition cannot be explained by the same plastic changes because the interval was too short to benefit from protein thesis. The reactivations, within this retention interval, likely promoted short-term synaptic facilitation mechanisms known to support working memory.^53^

The current reactivation findings have other important implications for understanding the neural organization of long-term memory. For instance, it has been strongly argued that memories initially dependent on the hippocampus, such as episodic memories, become independent of the hippocampus over time.^54–56^ Evidence supporting this argument comes from studies reporting temporally graded retrograde amnesia in patients with hippocampal lesions, meaning that recently, but not remotely, acquired memories are lost following the damage.^57,58^ Distributed Reinstatement Theory, however, suggests that time may not be the most critical factor involved in making memories independent of the hippocampus.^59,60^ With more remote memories, there are more opportunities for reactivations, and thus repeated bouts of reconsolidation which strengthen the memory beyond the hippocampus. Indeed, it has been shown that repeated distributed reactivations can preserve a memory following hippocampal damage,^26^ but not in all situations.^27^ Moreover, evidence from functional magnetic resonance imaging or functional studies in human participants suggests that repeated retrievals (reactivations) of a memory increase neuronal activity in areas aside from the hippocampus.^24,25^ Combined, the evidence from these studies suggest that reactivations extend the neural network supporting a memory, and arguably did the same for the image memories in the current study.

The complex features pertaining to the images we used provided the advantage of an in-depth recall assessment (e.g., gist vs. detailed information). The descriptive and component reactivations increased the number of images and details recalled, suggesting improved persistence/duration of the memory. The number of correct details recalled per image, however, is arguably a better measure of the qualitative properties (precision) of the image memories and only the descriptive reactivations improved performance on this index. Thus, the descriptive reactivations improved long-term memory for the gist as well as the content of the images. This is consistent with the evidence suggesting that more effortful and detailed recall shows better Testing Effect benefits,^41,44^ as well as the enhanced mnemonic precision reported in studies that examined the effects of reconsolidation in non-human animal studies.^27,28^ In contrast, the component reactivation strengthened the memory for the gist of the images. We cannot know how much of the target memory was reactivated in the component condition. However, because only one categorical feature was cued at the time of each reactivation, we can assume that the recall was partial and lacked detailed content. The finding that a memory can be strengthened without detail-specific enhancement may also lend support to the memory transformation view, which states that episodic memories become more semantically represented, at least in part, because of repeated reactivation of gist-like information.^61,62^

The reactivation benefits, whether from the Descriptive or Component group, were most prominent when contrasted with the Distractor group than the Control (no reactivation) group. The former provided a control for experimenter–participant interactions during the reactivation sessions. Perhaps reactivating irrelevant information of the experimental procedures added “noise” to the original image memories similar to memory updating, meaning the incorporation of new information into the target memory. But the absence of a statistical differences between the Distractor and Control groups as well as the null findings on the false memory measures fail to support this argument. Most importantly, the fact that the distractor reactivations did not strengthen the memory of the viewed images, but that the descriptive and component reactivations did, suggests that the reactivation content needs to pertain to the target memory in order to enhance retention.

In conclusion, we have replicated the Testing Effect through descriptive reactivations and, more importantly, we have shown that component reactivations, in which only categorical features are used to induce reactivation, can similarly mitigate forgetting. This suggests that an effortful description of the target memory is not critical to enhance retention, at least gist-like information retention. In addition, the findings suggest that memory reactivation, whether descriptive or component, leads to memory strengthening, a process commonly proposed within the memory reconsolidation literature.

## Methods

All procedures were approved by Trent University’s Research Ethics Board, which follows the guidelines of the Tri-Council Policy Statement of the Government of Canada.

### Participants

A sample of 183 participants from Trent University (156 female) with a mean age of 20.5 years (SD = 5.5) provide informed consent and completed this experiment. The participants were recruited via SONA, an online research participation system, and were given credit towards a psychology course as compensation. The data of 16 participants were excluded from the analyses due to issues with contacting the participants for the reactivation manipulation or the testing, resulting in a sample size of 167. This resulted in a group sample size ranging between 12 and 16, which is consistent with that of other studies in the field.^19,29^

### Materials

Twenty-six neutral images were selected from the IAPS. Specifically, all images ranged, according to IAPS, between four and six on the emotional valence dimension (M = 5.4, SD = 0.45) and below four on the emotional arousal dimension (M = 3.3, SD = 0.42). Nine images depicted landscapes, 3 were of animals, and the other 14 images were of household objects. The images were presented on a 15-in. Dell LED monitor, using a Dell computer and Microsoft PowerPoint 2013.

### Procedure

#### Acquisition phase

The participants were told they would view a series of images, and were asked to remember as much about each image as possible. The participants sat in front of, and approximately 60 cm away from, the monitor, and when ready, pressed the “enter” key on the computer keyboard to begin the experiment. The 26 images were presented individually for 10 s, with a black screen shown between each image for 3 s.

#### Reactivation manipulation

Each participant was randomly assigned to one of four different groups: Descriptive Reactivation (Testing Effect condition), Component Reactivation (Cueing Effect condition), Distractor, and Control (no reactivation).

The Descriptive Reactivation group received three separate memory reactivations by asking them to describe the images they had previously viewed. In each instance, they were asked the same question, “Please describe, in as much detail as you recall, the images you viewed in Part 1 (acquisition phase) of this experiment.” The aim of this question was to promote explicit effortful recall of detailed information of the images, similar to the process that is followed in studies examining the Testing Effect.^47^

The Component Reactivation group also received three separate memory reactivations, but by cueing a component/categorical feature of the images. In each instance, the participants needed to provide the number of images that included the component. The first question participants received was, “How many images included animals? Please do not describe the images, only state the number within the category,” question two, “How many images included landscapes? Please do not describe the images, only state the number within the category” and question three “How many images include household objects? Please do not describe the images, only state then number within the category.” Importantly, these reactivations did not require an explicit description of the target memory.

The Distractor group received three questions related to the experimental procedure which were not intended to trigger image reactivation. The objective of this condition was to provide an image-irrelevant reactivation control, meaning controlling for questioning of the participants during the retention phase. The first question was “What was the date and time when you completed Part 1 of this experiment?” question two, “What form did the experimenter go through with you before beginning the experiment?,” and question three “Please describe the procedure for Part 1 of this experiment.”

The Control group did not receive any questions throughout the period from viewing the images to completing the recall test. The objective of this control condition was to examine the typical rate of forgetting.

#### Retention interval

In addition to the reactivation condition, the participants were randomly assigned to one of three retention interval conditions: 1 h, 9 days, and 28 days.

##### 1-h condition

In the 1-h condition, the acquisition-to-test interval was approximately 1 h. Specifically, after viewing the images, the participants were given a 10-min break where they were provided reading material from magazines such as Psychology Today, Scientific American Mind, and New Scientist. Following the break, the participants responded to a question on the computer using Qualtrics survey software. The question differed according to the reactivation group to which the participant was assigned: Descriptive, Component, or Distractor. This was repeated another two times, again separated by a 10-min interval each time. After the third question, there was a final 10-min break before the retention test. Note that the participants in the Control group were not questioned and read for 50 min without interruption until the test.

##### 9-day condition

In the 9-day condition, the participants left the lab after viewing the images. Two days following the acquisition phase, the participants were sent a reactivation question via e-mail using the SONA system. The question differed according to the reactivation group to which the participant was assigned. The participants were given 12 h to respond to the question. If they did not respond within this period their data were excluded from the study. This procedure was repeated on the fifth and seventh day. Thus, the participant received a total of three equally spaced reactivations. Responses to all questions were recorded using the Qualtrics survey software. On the ninth day, the participants returned to the lab and completed a free-recall test. The participants in the Control group were not contacted during this period, with the exception of an attendance reminder e-mail sent the day prior to the free-recall test.

##### 29-day condition

The 28-day condition followed the same procedure as the 9-day condition, with reactivation questions received on days 7, 14, and 21. The participants returned to the lab on day 28 to complete the free-recall test. The participants in the Control group were not contacted during this period, with the exception of an attendance reminder e-mail sent the day prior to the free-recall test.

#### Retention test

Each participant completed a pen and paper free-recall test while seated at the location used in the acquisition phase. They were asked to recall as many images with as many details as possible. Specifically, the participants were asked “In part 1 of this experiment, you observed a series of images. In the space below, describe, in as much detail as possible, the images you recall.” The participants were given as much time as they required. Following completion of the test, the participants were debriefed on the purpose of the experiment.

### Indices of memory and statistics

The number of correctly recalled images, as well as the number of correct details listed for each image from the free-recall test were used as indices of memory. An image was considered correctly recalled if it described the gist of the image. A correct detail was tabulated using descriptors such as color, location, size, orientation, description, and items pertaining to the image. The number of falsely recalled images and the number of incorrect details were also tabulated to assess intrusions.

### Statistics

The data were trimmed by removing the scores (on each dependent measure) of the top two and bottom two participants on the correct image measure from each group. Trimming the tails of the distribution has been shown to produce more robust findings.^63^

The data were analyzed using a 3 × 4 between-subject ANOVA with interval conditions (1 h, 9 days, or 28 days) and reactivation groups (Descriptive, Component, Distractor, Control/no reactivation) as between-subject factors. LSD pairwise comparisons were also performed to examine specific group differences that followed the postulated hypotheses. An *α*-level of 0.05 was used in all instances.

### Data availability

All data collected for the current study are available, upon reasonable request, from the corresponding author.
